# Polyhydroxyalkanoates as multifaceted biopolymers: an eco-friendly alternative to conventional plastics

**DOI:** 10.1039/d6ra02201d

**Published:** 2026-06-01

**Authors:** Konjerimam Ishaku Chimbekujwo, Udeme Joshua Josiah Ijah, Oluwafemi Adebayo Oyewole, Olabisi Peter Abioye, Evans Chidi Egwim, Mattheus Victor Maso Lacorte Silva, Naga Raju Maddela, Jonas Contiero

**Affiliations:** a Department of Microbiology, Federal University of Technology Minna Nigeria; b Department of Microbiology, Modibbo Adama University Yola Nigeria; c Institute of Biosciences, Institute for Research in Bioenergy, Sao Paulo State University Rio Claro SP Brazil Konjechimbe@mau.edu.ng; d Department of Biochemistry, Federal University of Technology Minna Nigeria; e Departamento De Ciencias Biológicas, Facultad De Ciencias de la Salud, Universidad Técnica De Manabí Portoviejo-130105 Ecuador

## Abstract

Plastic pollution originating from fossil-based materials persists as a critical environmental concern due to their resistance to degradation, as well as their role in the build-up of microplastics and the release of greenhouse gases across waste-management processes. Polyhydroxyalkanoates (PHAs) are considered effective, biodegradable and bio-based alternatives. However, financial and process constraints limit the industrial-scale use of PHAs despite considerable investigations. Several bacteria can synthesise PHAs using different substrates and store them as granules under nutrient-limited conditions. Bacterial species such as *Bacillus*, *Pseudomonas*, *Halomonas* and *Cupriavidus necator* have been used to produce PHAs. This study provides an analytical and comprehensive evaluation of PHAs as multifaceted biopolymers, evaluating existing developments and highlighting major constraints. This review evaluates comparatively different feedstocks for microbial production and optimisation approaches, highlighting their effectiveness and limitations. It further evaluates developments in metabolic engineering, including CRISPR/Cas9-mediated genome editing systems, bridge recombination, and synthetic biological engineering, which aim to increase yield and modify monomer characteristics. Moreover, it evaluates downstream processing techniques, such as chemical, enzymatic, mechanical and biological extraction methods, emphasising their sustainability and scalability. Significantly, this review highlights key barriers to the large-scale production of PHA, such as high manufacturing costs, variation in raw materials, and operational limitations while suggesting a strategic pathway for future research. Finally, this paper offers an integrated viewpoint that connects the production, properties, and applications of PHAs, providing the understanding necessary to enhance their relevance as sustainable alternatives to conventional plastics.

## Abstract 2

Polyhydroxyalkanotes (PHAs) are biodegradable biopolymers produced through microbial synthesis, offering a sustainable alternative to conventional plastics with versatile properties and applications in agriculture, medicine and healthcare.

## Graphical abstract

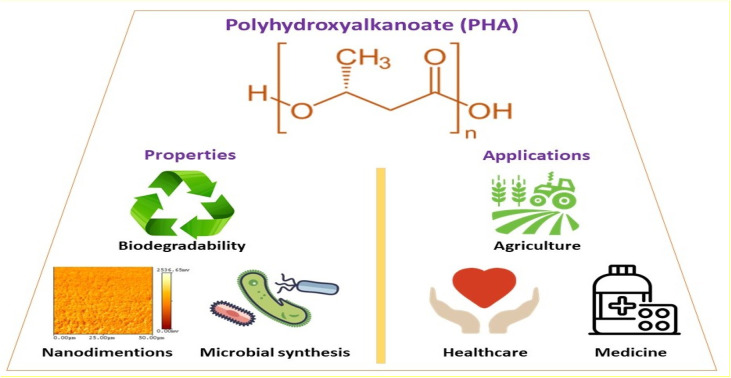

## Overview of plastic pollution, its ecological implications and sustainable alternatives

Traditional materials (*e.g.*, metals, glass, and wood) have been greatly replaced by petrochemical plastics since the 1960s due to their cost-effectiveness, durability, corrosion resistance, stiffness, low electrical and thermal conductivity, and lightweight nature. Based on these factors, plastics have been incorporated into modern life, with production exceeding 320 million tonnes globally in 2015 and reaching its peak at 400.3 million tonnes in 2022.^1,2^ Despite this rapid growth, only a small portion of plastics (9.6%) is derived from bio-based, biodegradable, or carbon-capture sources, highlighting a continued dependence on fossil resources. Production is projected to reach 460 million tonnes by 2030; concerns over sustainability and environmental impact are intensifying.^3^ However, specific properties that make plastics highly functional also contribute to their persistence in ecosystems. Traditional plastics resist degradation and eventually accumulate in terrestrial and marine ecosystems.^4,5^ As plastics degrade over time, they become microplastics and nanoplastics. These substances may negatively affect human health and ecosystems *via* bioaccumulation and lead to significant toxicity.^2,6^ Single-use plastic waste from food and beverages accumulates more rapidly, adding to worldwide plastic waste due to its high usage.^7^

The current management strategies for plastic waste, including landfill disposal, incineration, and recycling, are limited and inadequate. Landfill disposal is the most common waste management approach due to its ease and low cost. Nevertheless, plastic materials cause substantial environmental pollution in disposal sites, polluting streams, reservoirs, and seas, resulting in water contamination.^8^ Incineration breaks down of plastic wastes, reducing their size and generating energy. However, the benefit of waste burning is overshadowed by the emission of toxic secondary products that could cause air contamination,^9^ which poses significant health risks to nearby populations and contributes to broader environmental issues. Mechanical recycling, while eco-friendly, faces limitations due to the need for pure, uniform waste streams, often resulting in low-quality reprocessed products.^10^ Chemical recycling, by contrast, can process mixed plastics and recover monomers; nonetheless, it is energy-intensive and generates toxic by-products.^11^ A viable approach like pyrolysis promotes the conversion of plastic waste into energy sources; however, limitations associated with cost-effectiveness and environmental impacts persist, which raise concerns about its long-term sustainability and viability as a widespread solution for plastic waste management.^12^

**Fig. 1:**
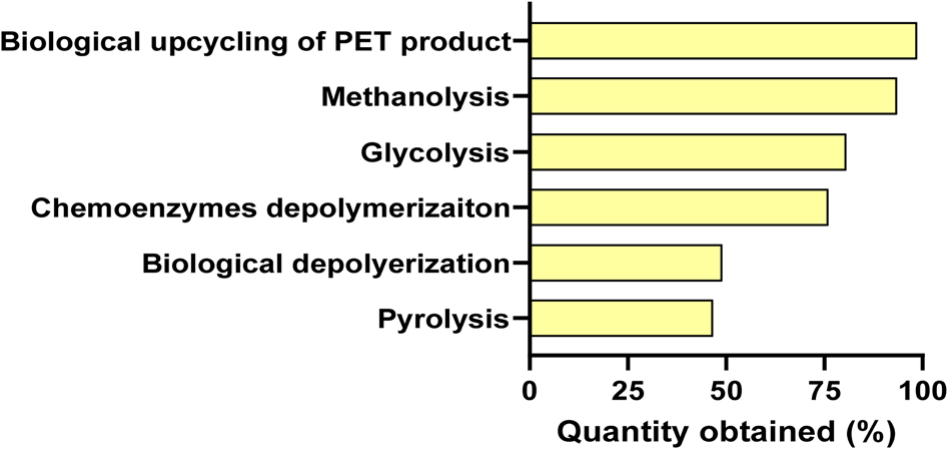
Methods of PET and PET product degradation [figure drawn using data from ref. 14–16].

To address these challenges, growing interest has moved toward plastic waste conversion, a method that converts synthetic plastic waste into value-added products instead of conventional disposal methods.^13^ Of the diverse types of plastics, polyethylene terephthalate (PET) has become a principal model material, owing to its broad utilisation, prevalence in discarded materials, and comparative ease of breakdown.^14^ Employing chemical methods, like methanolysis and glycolysis, PET can depolymerise to useful intermediates, for example, terephthalic acid (TPA) and ethylene glycol (EG). These products offer a critical connection between plastic waste and bioconversion routes.^15^ The latest development has revealed that PET-based monomers can be exploited as feedstock for microorganisms, allowing their transformation into valuable compounds, as shown in Fig. 1.^16^ In particular, combined chemical-enzymatic and biological approaches have established the conversion of terephthalic acid (TPA) and ethylene glycol (EG) into key metabolic intermediates, which can subsequently be channelled into the production of biodegradable biopolymers.^17^ This approach constitutes a substantial change from traditional recycling methods to a sustainable bioeconomy in which plastic materials act as a valuable resource instead of a pollutant. One of the most promising groups of materials generated through these pathways is PHAs, a group of biodegradable and biocompatible biopolymers biosynthesised by microbes. PHAs possess characteristics similar to those of petroleum-based plastics while offering the advantage of eco-friendly biodegradability. Significantly, recent findings have revealed that microbes like *Ideonella sakaiensis* and *Pseudomonas* sp. can degrade and assimilate PET-derived compounds for PHA production, thus establishing a direct relationship between plastic-waste upcycling and eco-friendly polymer synthesis.^18,19^ Consequently, this present review employs a holistic approach that integrates plastic-waste pollution, waste utilisation approaches and PHA biosynthetic processes within an integrated approach. Following the route from plastic-waste production *via* PET breakdown to microbial bioconversion into PHAs, this review highlights the capability of converting environmental pollutants into eco-friendly materials. This strategy not only addresses the shortcomings of conventional recycling processes but also highlights the potential of PHAs as sustainable, eco-friendly alternatives to conventional plastics.

## Alternatives to petroleum-based polymers

In recent times, there has been growing interest in producing eco-friendly substitutes to fossil-based plastics. Among these options, bioplastics obtained from sustainable feedstocks have attracted considerable interest due to their reduced ecological footprint and capacity for biodegradation. In particular, PHAs, a group of naturally occurring polymers produced by diverse prokaryotic and eukaryotic microorganisms, have gained recognition as strong alternatives to traditional plastics.^2^ PHAs exhibit multiple beneficial characteristics, such as biodegradability and biocompatibility, and they originate from renewable substrates, which allows for a broad spectrum of uses. Besides their physicochemical properties, PHAs provide significant environmental advantages. Researchers have linked their synthesis and utilisation to a substantial decrease in reliance on fossil fuels compared to petrochemical plastics.^20^ However, regardless of their benefits, widespread industrial implementation of PHAs continues to be limited by operational efficiency and scale-up potential, which are critical factors that hinder their competitiveness against traditional petrochemical plastics, particularly in terms of cost-effectiveness and production speed. This review, therefore, utilises a systematic and comprehensive approach for examining PHAs as environmentally sustainable substitutes for fossil-based plastics. Specifically, it analyses the production of PHA among diverse microorganisms; approaches to improving production efficiency *via* process optimisation and metabolic engineering; and developments in industrial-scale production, recovery, and purification. In addition, this review identifies major constraints on existing production processes and discusses developing strategies like waste volatilisation and advanced biotechnological approaches.

## Overview of PHAs

Polyhydroxyalkanoates (PHAs) are intracellular storage polymers synthesised by bacteria and archaea, existing as hydrophobic granules (0.2–0.5 µm) surrounded by a protein layer.^21^ They primarily serve as carbon and energy reserves during nutritional deficiencies, especially nitrogen deficiency, while enhancing microbial adaptation to stress.^22^ Bioplastics are a broad category of materials that can display bio-based, biodegradable, or both characteristics. It is important to note that not all bioplastics are derived from renewable resources, nor are all bio-based plastics biodegradable. Based on these properties, bioplastics are generally categorised into the following groups: bio-based and non-biodegradable plastics (such as polyethylene (PE), polypropylene (PP), polyamides (PA), and polytrimethylene terephthalate (PTT)); fossil-based and non-degradable plastics (including polyethylene terephthalate (PET), polyethylene (PE), and polypropylene (PP)); bio-based and biodegradable plastics (like polylactic acid (PLA), polyhydroxyalkanoates (PHA), starch blends and polybutylene succinate (PBS)) (although PBS are either bio-based biodegradable or fossil-based biodegradable); fossil-based biodegradable plastics (polybutylene adipate terephthalate (PBAT), polycaprolactone (PCL)).^23,24^

This classification emphasises that specific biodegradable polymers, like PBAT and PBS, can be produced from fossil-derived feedstocks, despite being able to undergo microbial breakdown under suitable conditions. These materials are broadly employed for applications like film packaging due to their desirable mechanical properties and ease of fabrication. Alternatively, certain bio-based plastics, like bio-PET (bio-based polyethylene terephthalate), are not easily biodegradable. In this context, PHAs are highly promising, as they are entirely bio-based and biodegradable, setting them apart from numerous other bioplastics.^25,26^ In contrast, PLA, while being bio-based, generally requires industrial composting environments for effective degradation.^23^ The global bioplastics market is composed of several major material categories. PHAs' current production remains relatively limited compared to more established bioplastics. For instance, polylactic acid (PLA), starch-based blends, and bio-based drop-in polymers (*e.g.*, bio-PE and bio-PET) currently prevail in the market due to their established developed industrial base and reduced cost.^24^ Structurally, PHAs consist of (R)-hydroxyalkanoic acid monomers, with side chain variation conferring high diversity.^27^ They are classified into short-, medium-, and long-chain types, with molecular weights ranging from 50 to 100 kDa depending on the producer strain.^28^ PHAs display unique mechanical and thermal properties. PHB is the most widely studied PHA, a rigid and brittle material with low flexibility, whereas copolymers like PHBV demonstrate enhanced elasticity and toughness.^29^ Emerging progress has facilitated the synthesis of more complex copolymers, including terpolymers composed of multiple monomer units. These developments have greatly broadened the spectrum of material properties, enabling PHAs to be customised for targeted applications and endowing them with advantageous functional properties, like biocompatibility, ultraviolet resistance, and barrier performance. These properties render them suitable for use in biomedicine, manufacturing industries and packaging.^30^ Despite these advantages, PHAs are strongly influenced by factors including substrate type, microbial strain, and enzymatic specificity,^31^ highlighting the need for process optimisation and system integration. Although ongoing developments continue to enhance their performance and versatility, limitations associated with production cost, scalability, and material uniformity continue to restrict their broad adoption. The multifunctionality of PHAs positions them as strong candidates to replace traditional petroleum-derived plastics; however, addressing existing economic and technical challenges remains crucial for their industrial-scale deployment. The following sections address these constraints and underscore the need for improved production strategies.

### PHA discoveries

The first documented report on the visualisation of PHA granules under the microscope was by Martinus Beijerinck in the year 1888. More examination was conducted on such cytoplasmic granules, which were initially identified as fats.^32^ A key finding was achieved in the year 1923, at which time Maurice Lemoigne revealed that polyhydroxybutyrate was produced by *Bacillus megaterium* during nutrient-deficient supply and without oxygen.^33^ Lemoigne, universally considered the pioneer of PHA studies, further examined polyhydroxybutyrate in depth from 1923 to 1951.^34^ Regardless of the initial breakthrough, investigations slowed down over the subsequent 30 years, primarily due to the ongoing debate over whether PHB was an intracellular storage compound or merely a by-product of metabolism. A key controversy prevailed: ‘Was PHB an intracellular storage compound? Or just a by-product?’ Lemoigne hypothesised an energy-storing function, but conclusive data were unavailable.^35^ The debate was clarified in the year 1958 upon the provision of proof by Macrae and Wilkinson, revealing that PHB decomposed in the absence of additional carbon input or buildup under elevated conditions of carbon-to-nitrogen ratios. Individual research shortly established PHB's metabolic function as an intracellular storage polymer.^36^ During this period in the 1950s and early 1960s, PHB was identified across various species of bacteria apart from the genus *Bacillus*; others are halotolerant bacteria and algal species.^33^

The 1970s marked a turning point in PHA research with the discovery of polymers distinct from PHB. While activated sludge technology had been developed in the UK in 1912 to treat wastewater, its potential for biopolymer production was recognised only six decades later, when Wallen and Davis proposed using activated sludge to generate PHAs for industrial applications.^37^ Although Li *et al.*^38^ are known to have used activated sludge to produce polysaccharides and PHB, a novel polyester analogous to PHB was discovered in 1972. The novel polymer was shown to be mainly composed of β-hydroxyvaleric (3-hydroxypentanoic) acid using nuclear magnetic resonance spectroscopy and infrared spectroscopy.^39^ This was the first report of a heteropolymeric PHA, establishing that PHB was not the only intracellular reserve biopolyester in bacteria. Following this breakthrough, a range of PHA copolymers were identified during the 1980s and 1990s,^40,41^ leading to their classification into short, medium, and long-chain-length types.^42^ Analytical techniques also advanced over this period. Early qualitative staining of PHB granules was achieved with Sudan dyes and later with Nile Blue A.^43^ Quantitative analysis initially relied on Lemoigne's gravimetric chloroform extraction method, which, although straightforward, lacks precision. This was later replaced by spectrophotometric assays in the 1960s.^44^ A major improvement came in 1978, when Braunegg *et al.* introduced a methanolysis method using sulfuric acid and chloroform, enabling PHB to be analysed as its methyl ester by gas chromatography.^45^ Compared to the earlier method, this technique cut down on mistakes in analysis and processing time, and it is still commonly used today with some improvements, such as enhanced sensitivity and automation in the gas chromatography process.^46^

### Types of PHA

PHAs are made up of monomeric components, such as 3-hydroxybutyrate (3HB), 3-hydroxyvalerate (3HV), 3-hydroxyhexanoate (3HHx), and 3-hydroxyoctanoate (3HO). Based on their monomer composition, PHAs are grouped into homopolymers and copolymers. Homopolymers are PHAs with repeating units, for example, PHB from 3HB and PHO from 3HO, while heteropolymers are PHAs with different monomers, for example, PHBHx from 3HB and 3HHx.^47,48^ PHAs are further classified into three groups: Those with 3 to 5 carbon atoms and short chain lengths (scl-PHAs) are called PHB and PHBV, produced by bacteria species such as *Cupriavidus necator, Burkholderia cepacia* and *Bacillus megaterium*.^49^ These PHAs show thermoplastic characteristics, but their fragility limits processability and hinders industrial usage. PHAs with 6 to 14 carbon atoms are medium-chain-length PHAs (mcl-PHAs), which include polyhydroxyolefin (PHO) and polyhydroxybutyrate-*co*-hydroxyhexanoate (PHBHx), and are produced by *Pseudomonas* species like *P. corrugata*, *P. aeruginosa* and *P. oleovorans*.^49,50^ In comparison to scl-PHAs, mcl-PHAs demonstrate more elastomer-like properties, are less crystalline, and are more flexible, which favor their use in applications requiring softness and elasticity. Nonetheless, their low tensile strength restricts their suitability in load-bearing applications. Long-chain-length PHAs (lcl-PHAs) have ≥14 carbon atoms, and oftentimes, microorganisms do not produce them; thus, they remain less explored due to biosynthetic limitations.^51^ Despite their potential for greater flexibility and distinct functional versatility, their limited accessibility and complex production processes restrict their industrial use.

### Microbial synthesis of PHA

PHAs are produced by various microbes as a survival mechanism to stress factors, most importantly, under limited nutrient conditions^52^ and in the absence of a surplus carbon substrate. This metabolic approach permits cells to accumulate excess carbon, consequently increasing viability. Significantly, PHA buildup can approach 94% (w/w) of the bacteria's dry cell weight,^32^ highlighting its relevance in carbon and energy storage. These polymers are accumulated inside the cell as lipid granules whose dimensions, number and stability are strictly controlled by linked proteins like phasins and phospholipids. PHA synthesis is mainly regulated by the primary carbon pathway and cell redox balance. In the pathway for PHA production, two molecules of acetyl-CoA condense to form acetoacetyl-CoA, which is further reduced to PHB to hydroxybutyryl-CoA in an NADPH-dependent reaction.^53^ This monomer is then polymerised by PHA synthase (PhaC) to form PHB granules. Notably, the yield of this pathway is strongly associated with the cell's NADH/NAD^+^ ratio, which controls carbon flow between the Krebs cycle and PHA synthesis. Besides scl-PHAs, microbes can synthesise mcl-PHAs *via* different metabolic pathways, especially *via* β-oxidation of fatty acids. In this route, enoyl-CoA hydratase (PhaJ) converts β-oxidation intermediates into (R)-3-hydroxyacyl-CoA monomers, which are further polymerised by PhaC.^54^ Moreover, the malonyl-CoA pathway offers a link between carbon metabolism and mcl-PHA biosynthesis. PhaG facilitates the conversion of fatty acid biosynthesis intermediates into (R)-3-hydroxyfatty acids, allowing the integration of compositionally diverse monomers into PHAs.^55^ Significantly, biosynthetic pathways are well documented; comparative contributions differ greatly depending on microbial type, carbon source, and growth conditions. This difference highlights a major challenge in this area: improving metabolic flows to obtain both high productivity and controlled properties for the polymer. Thus, recent studies have progressively focused on metabolic engineering to improve substrate availability and regulate monomer composition, which are crucial for large-scale production of PHAs.

### Enzymes specific to PHA production

The core enzymes of the PHA biosynthetic pathway, such as acetyl-CoA c-acetyltransferase (PhaA), acetoacetyl-CoA reductase (PhaB), and PHA synthase (PhaC), are encoded by the phaA, phaB, and phaC genes, respectively.^56^ In class-I PHA-synthesising microorganisms, the phaA, phaB, and phaC genes are arranged in the phaCAB operon, and their overexpression has been significantly influenced by the increased synthesis of PHA.^57^ In addition to these primary enzymes, phasins (PhaP, PhaI, PhaF, and others), many auxiliary proteins perform key functions in PHA granule formation, stability, and control. Phasins are proteins that maintain internal PHA granules and control their association with other intracellular structures. Regulatory proteins participating in PHA metabolic processes consist of transcriptional inhibitors like PhaR (PHA synthesis repressor) and PhaQ (PHB-responsive regulator), in addition to activators like PhaM, which jointly regulate the expression of metabolic genes depending on cellular and environmental factors.^58^ Moreover, degradative enzymes, including PhaZ (depolymerase) and PhaY (hydrolase), control PHA utilisation and recycling intracellularly.^58^ The equilibrium between PHA production and depolymerisation greatly affects overall polymer storage. For example, the knockout of the PhaZ gene in *P. putida* KT2442 has been reported to improve the storage of PHA, whereas simultaneous expression of PhaZ with PhaC can decrease the total polymer storage because of concurrent synthesis and breakdown processes. These results indicate that the suppression of PhaZ or the enhancement of PhaC can increase total productivity.^58^

Besides the primary metabolic pathway, the core carbon metabolism greatly affects PHA synthesis *via* the supply of essential precursors, like acetyl-CoA and pyruvate. Enzymes, including pyruvate dehydrogenase complex (PDH) and acetyl-CoA carboxylase (ACC), control metabolic flux toward PHA production.^59^ Acetyl-CoA supply is a critical constraint in PHA synthesis, and its metabolic distribution is essential in influencing production yield.^60^ When nutrients are abundant, increased CoA may inhibit PhaA function, thereby lowering polymer accumulation.^61^ Fatty acid biosynthesis is also connected to PHA synthesis. Enzymes like 3-oxoacyl-[acyl-carrier-protein] synthase (FabB), 3-oxoacyl-[acyl-carrier-protein] reductase (FabG), and [acyl-carrier-protein] S-malonyltransferase (FabD) participate in fatty acid biosynthesis and indirectly impact PHA synthesis by regulating the level of acetyl-CoA and associated intermediates. Moreover, alteration in β-oxidation pathways can change monomer composition and influence the material property of the produced PHA.^62^

### Microorganisms involved in PHA production

**Fig. 2:**
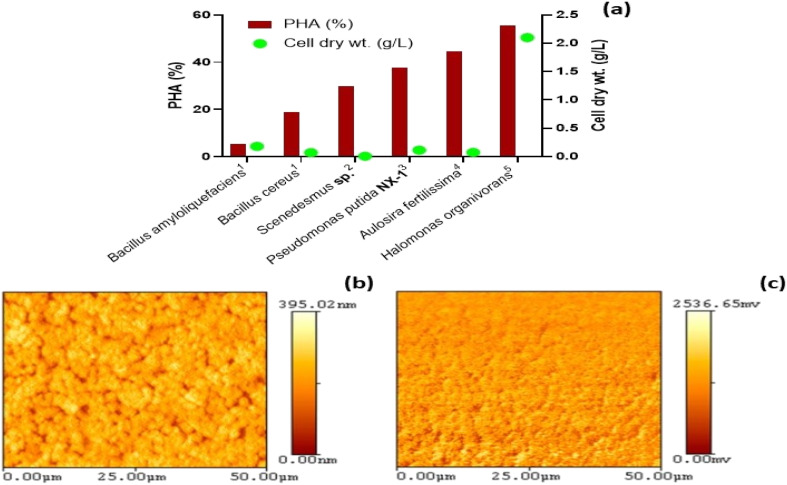
Microbial polyhydroxyalkanoate (PHA). (a) Bacteria-producing PHAs (in percent, left *y*-axis in vertical bars) and their cell dry weight (in g L^−1^, right *y*-axis in solid circles) [figure drawn using data from ref. 63–67]. Images of surface morphology acquired using atomic force microscopy in tapping mode for bacterial PHA thin films: (b) height and (c) demodulation 50 × 50 (µm) images [AFM images are from an open-access source, ref. 68].

Numerous microbial strains encompassing both Gram-positive and Gram-negative species, as well as select archaeal and microalgal species, have demonstrated the capability to synthesise PHAs. Within this group, the most extensively studied producers are *Bacillus megaterium*, *Ralstonia eutropha* (also known as *Cupriavidus necator*), and *Pseudomonas putida*. Other prominent PHA-producing bacteria include *Halomonas organicvora*, *Bacillus amyloliquefaciens*, *Bacillus cereus, Scenedesmus* sp*.,* and *Aulosira fertilissima*,^63–67^ which were also reported (Fig. 2). Representatives from the families *Azotobacter*, *Syntrophomonas*, *Aeromonas*, and *Clostridium* are also included.^69^ From a physiological standpoint, PHA-producing microbes can be divided into two main categories: (i) nutrient-limited producers, which store PHAs under conditions of carbon surplus and nutrient limitation (like nitrogen or phosphorus), for example, *C. necator*, *P*. *putida*, and *P. oleovorans*; and (ii) growth-associated PHA producers, which produce PHAs during active cellular proliferation, for instance, *E. coli* (engineered strains) and *A. latus*.^70,71^ This classification is particularly relevant in industrial applications since nutrient-restriction approaches typically necessitate dual-phase fermentation, thereby increasing process complexity and operational expenses. In contrast, growth-associated production provides opportunities for streamlined and continuous processing. Different species exhibit substantial variation in the type of PHA produced.^72^ For example, *C. necator* primarily synthesises short-chain-length (scl) PHAs, which are generally more rigid and brittle, while *Pseudomonas* spp.^73^ mostly synthesises medium-chain-length (mcl) PHAs with enhanced flexibility and elastomeric characteristics. This illustrates an important compromise between polymer properties and microbial performance, as strains that favour certain material characteristics may not necessarily exhibit high productivity or rapid growth rates. To mitigate the limitations of wild-type strains, genetic engineering has become an effective approach for increasing PHA yield.

Genetically modified systems, especially in *Escherichia coli*, have allowed the expression of PHA biosynthesis pathways, leading to polymer accumulation reaching up to 90% of cellular dry weight.^74^ The repression of the bceA gene using CRISPRi has resulted in complete suppression of EPS biosynthesis and a substantial increase in PHB yield. The ΔbceA mutant exhibited a 1.75-fold rise in PHB concentration and yielded polymers with higher molecular weight compared to the wild-type *Burkholderia* sp. SCN-KJ.^75^ This shows the benefits of repression route strategies in reducing substrate loss to competing metabolites. Similarly, the *Escherichia coli* DH5α cscBKAint::pBR1MC-2::phaCAB strain has achieved 47.3% ± 6.7% P(3HB) using molasses and a maximum tensile strength of 26.3 ± 2.1 MPa.^76^ In contrast, genome editing using a CRISPR-Cas9-nickase system to knock out single and double intracellular depolymerases (phaZ1 and phaZ2) has shown substantially improved PHA yield, using acetate, resulting in the highest PHA content (27.3 wt%) with a copolymer 3HV of 1.2 mol%, specifically when phaZ was deleted.^77^ In contrast, genome editing using a CRISPR-Cas-based editing tool to delete the double mutant (ΔPS1; ΔprpC) in *H. nigrificans* X339 resulted in a CDW of 12.91 g L^−1^, the highest PHA content of 62.5% for PHBV, a 7.57% monomer composition using propionate as a substrate, and a conversion rate of 100%.^78^ Such engineered strains provide multiple benefits, including high growth rates, increased PHA yield, improved polymer quality,^79^ well-understood genetic systems and the capacity to metabolise a wide range of inexpensive substrates, making production using microbial systems sustainable for large-scale biopolymer manufacturing.^74^ Nevertheless, they also present challenges associated with genetic instability, regulatory constraints and process robustness at an industrial scale. Looking at the sustainability perspective, microbial strain selection must be evaluated in conjunction with substrate utilisation. Although *Escherichia coli* and *Cupriavidus necator* exhibit high efficiency on purified substrates, their efficiency may be reduced when grown on complex and variable waste-derived substrates. Conversely, extremophiles, such as *Halomonas* spp. and *Haloferax mediterranei*,^75^ demonstrate enhanced tolerance to high salinity and contaminants, making them better suited for the use of industrial and saline waste streams. These properties minimise the requirements for sterile conditions and stringent process control, thereby reducing overall production costs. In addition, the selection of durable and low-maintenance microbes that can utilise locally sourced waste feedstocks is significant. Microbes capable of functioning under non-sterile fermentation conditions and metabolising mixed feedstocks provide a feasible route for distributed and economically viable PHA production systems.^41^ No individual microbial system presently meets all the criteria for efficient, scalable, and sustainable PHA synthesis. As such, future studies should thus prioritise engineering resilient and adaptable microbial consortia capable of metabolising heterogeneous waste feedstocks, combining microbial engineering with process optimisation for industrial-scale implementation, improving genetic stability and ensuring regulatory compliance of engineered strains. These integrated strategies are critical to closing the gap between laboratory-scale research and the industrial-scale commercialisation of PHAs.

### Substrates employed for PHA production

**Fig. 3:**
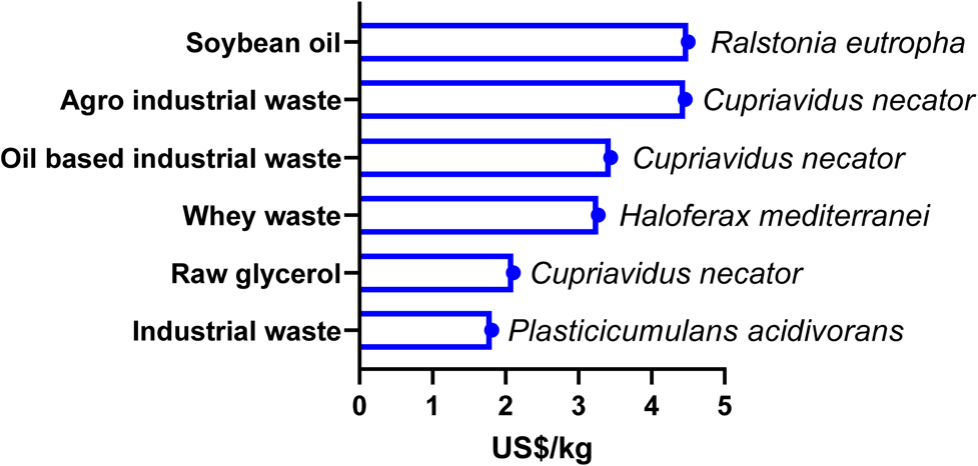
Maximum unit cost (US$ per kg) of producing polyhydroxyalkanoate from different feedstocks using different microbial species [figure drawn using data from ref. 86–91].

Substrate selection is a very crucial factor in PHA production, impacting not only the economic efficiency of the process but also the physicochemical characteristics of the final polymer. Substrates can contribute up to 50% of the overall cost of production,^80^ highlighting their importance as a key determinant in the commercial feasibility of PHA-derived bioplastics. Moreover, diverse carbon substrates are metabolised *via* distinct metabolic pathways, giving rise to variations in monomer compositions, copolymer structures, and material characteristics.^81,82^ PHA production substrates are broadly categorised into five groups: gases, *n*-alcohols, carbohydrates, *n*-alkanoic acids, and *n*-alkanes. Of these carbon sources, including glucose, sucrose has traditionally been utilised due to its high bioconversion yield and well-characterized microbial metabolic routes. Yet its utilisation poses notable ethical and economic concerns since it directly competes with food and feed resource usage.^83^ This food *versus* material challenge has led to the adoption of more eco-friendly alternatives. In the last decade, second-generation and waste-derived substrate feedstocks have received growing attention as low-cost and environmentally sustainable substrates. They consist of whey, glycerol,^84^ lignin,^85^ wastewater, industrial waste, used coffee grounds, starch residues, and wheat and rice straw.^80^ Agro-industrial waste (molasses), raw glycerol (88% wt) processed from crude glycerol, oil-based industrial waste (NCIMB), industrial waste (paper mill/food), soybean oil and whey waste have been used for large-scale production of PHA by *Cupriavidus necator*, *Plasticicumulans acidivorans*, *Ralstonia eutropha* and *Haloferax mediterranei*, with unit production costs of 4.46 $ per kg, 2.108 $ per kg, 3.44 $ per kg, 1.81 $ per kg, 4.5 $ per kg and 3.27 $ per kg,^86–91^ respectively, as shown in Fig. 3. Despite their cost-effectiveness and environmental sustainability, they exhibit notable constraints; for instance, plant-derived lignocellulosic substrates can potentially decrease process yield.^92^ In a similar manner, unrefined and variable waste streams may introduce contaminants that potentially impact microbial growth, biopolymer yield, and downstream processing steps.^80^ This issue becomes critical in premium applications, such as in medicine, where high polymer purity is paramount.^41^

An important aspect involves the compromise between cost savings and product uniformity.^93^ Although waste-derived feedstocks reduce production expenses, they commonly cause inconsistencies in polymer structure, molecular weight, and physicochemical properties because of the heterogeneous feedstock composition.^81^ Such variability represents a significant challenge in commercial-scale production where consistent quality and reproducibility are necessary. Recent studies have evaluated alternative carbon sources, including volatile fatty acids (VFAs) and intermediates obtained from plastics (ethylene glycol), which offer potential pathways to connect waste upcycling with biopolymer production. For example, VFAs significantly promote PHA production by *Cupriavidus necator*,^27^ while genetically modified *P. putida* (KT2440) can metabolise ethylene glycol to produce medium-chain-length PHAs in regulated experimental settings.^94,95^ These methods demonstrate the possibility of integrating plastic recycling with PHA biosynthesis within the context of a circular bio-based economy.

Substrate selection needs to be considered not only with respect to production yield but also to process sustainability, scale-up potential, and regional resource availability.^71^ In low- and middle-income regions, agro-industrial residues are widely available, yet technological infrastructure is underdeveloped. Using locally affordable feedstocks is a feasible route for decentralised PHA production. However, this approach necessitates the engineering of robust microorganisms, along with simplified processing methods capable of handling variable feedstock compositions,^31^ which are essential for ensuring consistent PHA production and overcoming challenges related to feedstock variability. In summary, despite notable progress in expanding the range of feedstocks for PHA synthesis, further research should concentrate on integrated criteria for affordability, high productivity, uniformity, and scalability, as well as enhancing the use of mixed and waste-based substrates, increasing microbial resistance to contaminants, coupling pretreatment and bioconversion processes,^41^ and designing economically viable downstream processing and purification methods. Such initiatives are vital to address existing limitations and facilitate the industrial-scale implementation of PHAs as eco-friendly substitutes for traditional petroleum-based plastics.

### Fermentation processes used to produce PHAs (conditions)

Three common fermentation strategies for bacterial PHA production are batch, fed-batch, and continuous processes. Although each strategy aims to maximise important variables, such as temperature, pH, fermentation time, and the carbon-to-nitrogen ratio, their efficiencies differ significantly.^96^

#### Batch cultivation

Batch fermentation is the easiest and most used method, in which the carbon source and other culture media components are supplied at the beginning of the process without addition and are not removed during cultivation. PHA is recovered from the accumulated biomass after cells are harvested at the end of the run.^97^ It can operate as a one-stage process, where biomass formation and PHA accumulation occur simultaneously in a closed system, or as a two-stage process, where biomass formation is followed by a dedicated PHA accumulation phase.^31^ Despite batch fermentation's operational simplicity and cost-effectiveness, it presents challenges of substrate depletion and metabolite accumulation. These limitations, such as carbon-source depletion, often trigger intracellular depolymerisation of PHA, as the polymer metabolises to serve as carbon and energy for cell maintenance, reducing overall yield.^98^ For example, the cultivation of *Azohydromonas australica* DSM 1124 on sugar in batch culture yielded 8.71 g L^−1^ CDW with 6.24 g L^−1^ PHB after 36 h, corresponding to a modest growth rate of 0.17 g L^−1^ h^−1^, reflecting modest productivity. When compared with other approaches, batch systems provide minimal control over substrate availability and metabolic flow, which limits their productivity and consistency. Therefore, while it is well-suited for lab-scale experiments, batch fermentation is commonly regarded as less favourable for large-scale industrial PHA production where increased yields and process robustness are required.^31^

#### Fed-batch cultivation

Fed-batch fermentation is broadly recognised as a more effective strategy for PHA production when compared with batch fermentation, largely attributed to its capability to control nutrient availability and prevent carbon limitations during cultivation.^99^ By allowing regulated nutrient additions, this process preserves favourable growth conditions, achieves higher cell densities, and improves PHA accumulation.^100^ Unlike batch fermentation, where carbon depletion and physiological imbalances limit yield. Fed-batch systems permit flexible regulation of metabolic flux, thus enhancing production efficiency and operational stability.^101^ Nevertheless, this enhanced productivity entails increased process complexity. Real-time monitoring and adjustment of substrate concentration remain challenging and can increase overall cost, especially at an industrial scale, due to the need for sophisticated sensors and control systems to maintain optimal conditions throughout the fermentation process. Notwithstanding these limitations, when optimised, fed-batch fermentation provides highly consistent results and is more appropriate for commercial use than batch methods.^101^ Quantitative data demonstrate the merits of this approach. For instance, wheat straw hydrolysate was employed for PHB production, yielding a biomass and PHA concentrations of 135.8 g L^−1^ and 105.0 g L^−1^, respectively,^102^ which are substantially higher than conventional batch yields. Similarly, high-cell-density cultures reported up to 71 g L^−1^ biomass, 56% PHA content, and a productivity of 1.44 g L^−1^ h^−1^.^103^ In another study, Pseudomonas putida KT2440 cultivated on oleic acid reached 141 g L^−1^ CDW and accumulated 72.6 g L^−1^ mcl-PHA within 38 hours, corresponding to a productivity of 1.91 g L^−1^ h^−1^.^104^ Overall, these results underscore that fed-batch fermentation delivers significantly higher yields and productivities compared to batch systems, making it far more suitable for industrial-scale PHA production. However, cost-effectiveness relies on optimising/increasing yield to offset the elevated costs related to operational control and process monitoring.

#### Continuous cultivation

In contrast to batch or fed-batch techniques, continuous fermentation creates a chemostat culture by preserving ideal conditions for biomass development and PHA accumulation.^105^ In this system, PHA-rich cells are harvested continuously, allowing for the steady production of polymers with consistent yield and quality.^46^ Compared to batch fermentation, this approach offers the advantages of reproducibility and process stability; however, prolonged operation poses challenges that limit large-scale adoption. Genetic instability and contamination of the production strain can happen in continuous systems, which could make them less efficient over time,^40^ potentially leading to decreased polymer yields and increased costs associated with strain maintenance and quality control. Moreover, constant production of residual broth in large quantities requires effective downstream processing and recycling approaches, leading to higher operational complexity and expenses, particularly in managing the separation and purification of the desired products from the residual biomass and by-products. From a metabolic standpoint, continuous cultivation could be of advantage for nutrient-restricted conditions that enhance polyhydroxyalkanoate (PHA) production. For instance, *P. oleovorans* ATCC 29347 has demonstrated the ability to produce mcl-PHAs under carbon-rich conditions and nitrogen deficiency, with constant nitrogen levels and increasing carbon availability enabling sustained PHA synthesis.^42^ On the other hand, in comparison with fed-batch systems, continuous systems frequently face challenges attaining equivalent cell densities and total productivity because of dilution effects and the necessity for rigorous control, which can lead to lower overall yields and increased operational costs. Continuous cultivation provides conceptual benefits of stable operating conditions, consistent product quality and uniformity. Operational limitations regarding control of contamination, strain stability, and downstream recovery processes presently limit the large-scale implementation of commercial PHA production, which hinders the ability to achieve the desired efficiency and cost-effectiveness in production compared to other methods.

### Enhancing PHA production

#### Process optimisation using statistical approaches

Traditional methods for optimising multivariate fermentation processes are labour-intensive, time-consuming, and ineffective, especially when addressing intricate interactions among factors.^106^ The traditional “one variable at a time” approach does not adequately account for such interactions and generally necessitates numerous experimental runs, making it inefficient for an effective optimisation process.^107^ To mitigate these challenges, statistical methods, like the response surface methodology (RSM), have been broadly applied to optimise PHA. RSM facilitates the concurrent assessment of multiple factors, like substrate concentration, pH and temperature, while revealing interactions among these variables and others.^108^ Using regression models, analysis of variance, and visualisation tools, such as contour plots and response surfaces, RSM offers a structured and systematic framework for identifying optimal operational conditions while minimising the number of experimental trials.^109^ Multiple studies have shown the efficacy of RSM in increasing the PHA yield. For instance, culture parameter optimisation produced a fivefold rise (19.5 g L^−1^*vs.* 3.7 g L^−1^) in PHA production by *Wickerhamomyces anomalus* VIT-NN01 utilising sugarcane molasses as a substrate.^110^ Likewise, increased PHA yield has been observed using *Burkholderia cepacia* BPT1213, *Pandoraea* spp. ISTKB and *Acinetobacter junii*, with notable improvements in both cell biomass and polymer accumulation.^111–113^ High levels of PHA production have also been achieved under optimised conditions, including polymer contents reaching up to 94% in *Enterobacter cloacae* SU-1 and 50% PHB in *Bacillus cereus* CCASU-P83.^114,115^ Although these results are promising, RSM has certain inherent constraints. It is primarily useful for fine-tuning existing process variables rather than significantly enhancing microbial efficiency or substrate utilisation. Moreso, models established under controlled laboratory settings may not reliably forecast performance at an industrial scale, where fluctuations in feedstock composition, contamination risks, and process variability are more significant. From a broader perspective, although RSM is an effective tool for enhancing process efficiency and minimising experimental effort, it should be combined with complementary approaches like metabolic engineering, the development of resilient strains, and process intensification techniques to enable cost-effective and scalable PHA production (Table 1).^116^

**Table 1:** Optimization of PHA production using surface response methodology

PHA type	PHA content/concentration	Conditions	Bacteria	References
PHA	57.9%	Inoculum size 8% (v/v), temperature 25 °C, incubation time 48 h	*Bacillus cereus*	117
PHB	76.2%	Inoculum size, 10% (v/v), temperature of 37 °C, incubation time 72 h	*Bacillus megaterium pPSPHAR1/1*	118
PHB	5.94 g L^−1^	Inoculum size 1.74, (NH_4_)_2_HPO_4_ concentration 1.0, pH 6.37	*Halomonas meridiana*	119
PHB	72.96%/6.78 g L^−1^	Temperature 28 °C, inoculum size 2.5% (v/v), 5 days	*Ensifer* sp*. Strain HD34*	120
PHB	66.07%/3.35 g L^−1^	1.5 v/v, 300 rpm, and 72 h	*Burkholderia cepacia BPT1213*	121
[P(3HB-*co*-3HV)]	79.68%	Incubation time (96 h), pH (10), temperature (37 °C)	*Pichia kudriavzevii VIT-NN02*	110

#### Genetic and synthetic biology approaches for enhancing PHA production

Enhancement of PHA can be substantially increased by genetic and metabolic engineering approaches targeted at increasing cell density, polymer accumulation, and overall bioprocess efficiency.^116^ Early advancements confirmed the viability of this approach when PHA biosynthetic genes from *Alcaligenes eutrophus* were first cloned into *E. coli* in 1987, leveraging *E. coli*'s rapid growth and efficient cell disruption for polymer recovery.^122^ Thereafter, engineered *E. coli* strains have been extensively used, achieving polymer contents of around 70 wt% in comparison to levels reaching up to 90 wt% with native producers, like *A. eutrophus*.^123^ This reveals a critical balance between ease of manipulation and peak production capacity. Metabolic engineering has additionally facilitated the channelling of carbon flux toward PHA synthesis by increasing precursor availability and reducing competing metabolic routes.^124^ This strategy enhances production yield and enables precise control of polymer characteristics, including flexibility, elasticity, and thermal stability, *via* the regulation of monomer composition.^125^ Furthermore, the incorporation of heterologous biosynthetic pathways has facilitated the co-production of novel compounds alongside PHAs, highlighting the versatility of engineered microbial systems. Such modifications frequently impose a metabolic burden on the host cell, which may decrease growth efficiency and process stability on a large scale.^126^ Recent progress in genome engineering tools, such as the CRISPR/Cas9-mediated genome-editing system, has introduced robust tools for accurate and efficient modification of microbial genomes.^127^ These technologies facilitate targeted gene insertion, deletion, and regulation, thereby enhancing PHA production, altering the copolymer composition, and increasing intracellular storage capacity. For instance, CRISPR-based repression of key cell division genes has been reported to enlarge cell size and enhance intracellular PHA accumulation, while pathway-specific regulation enables controlled incorporation of monomers, such as 3-hydroxyvalerate.^128^ Despite these strengths, the implementation of CRISPR-based technologies at an industrial scale remains constrained due to concerns regarding genetic stability, regulatory requirements, and performance under fluctuating conditions, which can lead to inconsistent PHA production and potential regulatory hurdles that may delay commercialisation. Novel genetic engineering approaches, such as bridge recombination, provide further benefits by allowing the simultaneous modification of multiple genes in a single step, thereby minimising the time and complexity involved in strain development. In comparison to CRISPR/Cas systems, which depend on DNA cleavage and repair processes, bridging recombination enables more seamless gene integration, deletion, or replacement.^41^ This strategy shows potential for accelerating the development of high-efficiency PHA-producing strains with enhanced polymer characteristics, like improved thermal resistance, tensile strength, and biodegradability.^41^ Nonetheless, its application is still at an early stage, and additional validation is necessary to evaluate its scalability and robustness. Concurrently, synthetic biology strategies have provided novel solutions to minimise downstream processing expenses, which constitute a significant barrier to the commercialisation of PHAs. One such approach entails the engineering of microbial systems to directly secrete PHAs, thereby removing the need for cell lysis and complicated extraction procedures, making it highly suitable for industrial-scale applications.^80,129^ Despite these developments, several significant challenges persist. Engineered strains frequently display decreased robustness when exposed to waste-based feedstocks, and sustaining genetic stability during extended industrial fermentation is challenging. The integration of advanced genetic technologies with cost-effective, large-scale production systems continues to be a significant bottleneck. Although genetic and synthetic biology strategies have greatly improved the efficiency and flexibility of PHA production,^129^ their effective industrial application will depend on achieving a balance between high productivity, robustness, economic viability, and scalability. Hence, further research should focus on developing stable, high-efficiency strains capable of efficiently metabolising heterogeneous feedstocks under practical industrial conditions.

#### PHA extraction and purification techniques

The extraction of PHAs is a major cost-limiting step in their commercial-scale production, which accounts for about 30% of total production costs.^130^ Extraction methods are generally categorised into chemical and physical methods. These can be used alone or in combination to enhance recovery efficiency.^131^ There are four main steps to recovering PHAs from fermentation: (1) biomass separation, (2) biomass pretreatment, (3) PHA extraction, and (4) polymer purification. The effectiveness of these stages is largely governed by processing conditions and the physiological condition of the culture, with efficient recovery commonly occurring during periods of high polymer storage within cells.^132^

#### Separation of biomass

**Fig. 4:**
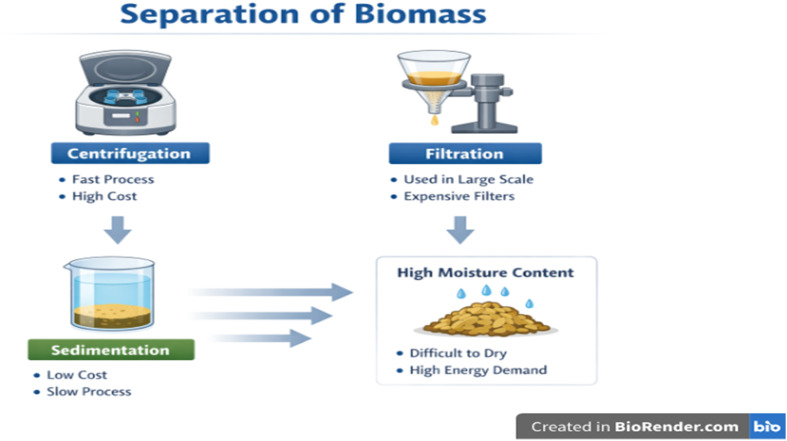
Separation techniques for cell biomass.

Cell separation represents the initial phase of PHA extraction and plays a key role in affecting the overall process performance. Frequently used techniques include centrifugation and filtration, both of which are extensively used in lab and industrial-scale systems, as shown in Fig. 4.^133^ Centrifugation provides rapid and efficient biomass recovery, rendering it appropriate for controlled conditions. However, it is energy-demanding and may not be cost-effective or viable for industrial-scale processes, particularly when compared to other methods like filtration and sedimentation that may offer more economical alternatives. Alternatively, filtration is better suited for large-scale operations but demands a specific membrane with precise pore dimensions, increasing production costs.^134^ Sedimentation has been examined as a cheaper option; nonetheless, low processing speeds reduce its usefulness in rapid production operations. Moreover, recovered biomass contains significant residual moisture, demanding energy-consuming drying steps before extraction, which further increases the total production costs.^133,134^ These challenges emphasise the importance of more effective and affordable recovery technologies, especially for industrial-scale PHA manufacturing.

#### Pretreatment

Pretreatment is a key step in PHA extraction, as it improves membrane permeability or promotes cell lysis, thus improving extraction yield. Despite these advantages, selecting a pretreatment technique greatly impacts the total production cost, industrial scalability, and polymer quality, making it a major optimisation step in PHA production processes. Various pretreatments have been examined, each presenting unique benefits and drawbacks. Lyophilisation (freeze-drying) is very effective in removing intracellular water while preserving biomolecular integrity, thereby reducing polymer degradation. Notwithstanding, this approach has high processing costs and energy consumption, which restrict its use in commercial-scale production,^135^ particularly in industries where cost efficiency is crucial, such as pharmaceuticals and food preservation. Likewise, the freezing method, whether gradual or rapid (*e.g.*, in liquid nitrogen), can preserve cell structure, with rapid freezing minimising ice-crystal formation, thereby reducing cell damage.^136^ However, these methods are largely limited to laboratory use rather than commercial-scale production. Salt-mediated lysis provides a cost-effective option, where elevated salt levels break down cell membranes and enhance PHA recovery. Although cost-effective, this method can lead to downstream processing constraints related to the removal of residual salts and the final product quality, which may affect the overall efficiency and viability of the PHA recovery process. Thermal pretreatment approaches are more extensively used in large-scale operations, owing to their simplicity and ease of scale. Lower temperatures aid in intracellular moisture removal, while higher temperatures promote cell rupture. Infrared heating induces molecular vibration and surface evaporation but has limited penetration, requiring a large surface area. Hot air drying improves evaporation efficiency; however, it also incurs higher operational costs. Drying at 45 °C produces results comparable to lyophilisation, whereas 60 °C and 80 °C further enhance drying efficiency.^137^ Pressurised hot water treatment is considered a more sophisticated method, integrating heat and pressure effects to disrupt cells using pressure while dissolving non-PHA materials, yielding a PHA-enriched hydrophobic phase.^138^ Generally, pretreatment methods can substantially improve recovery efficiency; however, their selection involves compromises between economic cost, energy consumption, scale-up potential and product quality. This emphasises the need for combined pretreatment methods tailored to specific production processes.

## Extraction

**Fig. 5:**
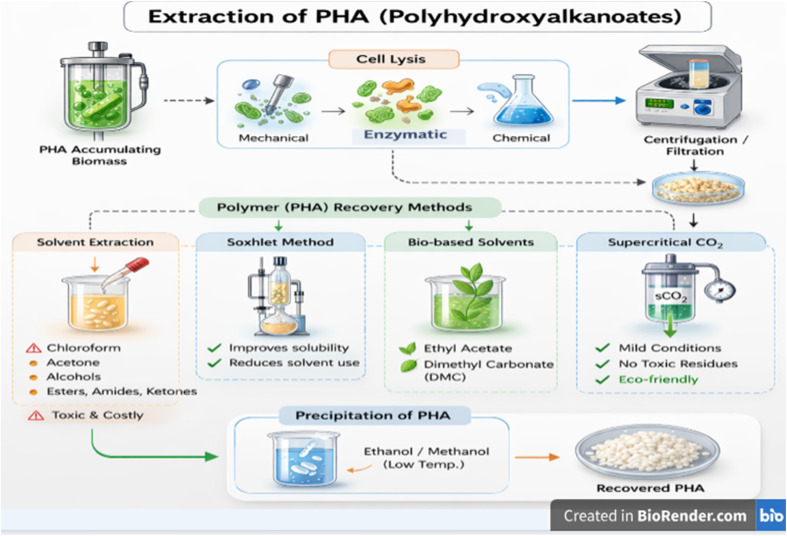
Polyhydroxyalkanoate (PHA) extraction methods.

PHA recovery can be accomplished by solubilising the biopolymer or removing cellular impurities after a cell disruption. Cell lysis can be accomplished using mechanical, enzymatic, or chemical methods, frequently combined with downstream recovery techniques, like centrifugation or filtration. Of these methods, solvent extraction remains the predominant technique because of its high efficiency. Solvent extraction is based on the selective dissolution of PHAs in selected organic solvents, which diffuse through cell membranes and solubilise intracellular PHAs. Polymer precipitation using non-solvent agents like ethanol or methanol facilitates polymer recovery, as shown in Fig. 5.^46^ Halogenated solvents, particularly chloroform, are frequently used due to their efficiency; however, their high cost, toxicity, and environmental hazards significantly limit their large-scale application.^139^ Process enhancement techniques, including Soxhlet extraction, improve solvent efficiency and reduce solvent consumption,^140^ but do not completely resolve environmental issues.

To address these challenges, substitute solvents, like acetone, alcohols, esters, amides, and ketones, have been evaluated, notably for mcl-PHAs. Despite being less harmful than halogenated solvents, many still present environmental and safety risks, highlighting the ongoing balance between recovery efficiency and environmental sustainability.^72^ More recently, bio-derived solvents like ethyl acetate and dimethyl carbonate (DMC) have been proposed as more sustainable options, providing lower toxicity and enhanced environmental compatibility.^141^ Moreover, their industrial feasibility remains uncertain. Supercritical fluid extraction, particularly using supercritical carbon dioxide (CO_2_), offers a potentially sustainable option. Based on its distinct physicochemical characteristics, supercritical carbon dioxide (CO_2_) integrates gas-like diffusion with liquid-like solvent capacity, facilitating efficient recovery under moderate conditions while eliminating harmful solvent residues.^142^ However, high initial investment costs and operational complexity of supercritical systems remain major limitations to their large-scale implementation, which could hinder the transition from solvent-based extraction to more sustainable methods, like supercritical fluid extraction. Solvent-based extraction remains predominant; increasing environmental and regulatory constraints are promoting the advancement of more sustainable alternatives, such as supercritical fluid extraction and other green chemistry methods that reduce environmental impact. Future research should prioritise the integration of cost-efficient processing strategies designed to achieve industrially scalable and environmentally sustainable PHA extraction.

## Methods of digestion

The recovery of PHAs is a major determinant of the total production cost and material quality, and thus, it poses a key challenge to large-scale production.^134^ Recovery strategies have been proposed, generally classified into chemical, enzymatic, biological, mechanical, and cell fragility-based approaches, each with inherent strengths and limitations. Chemical digestion remains one of the most used methods due to its ease of operation and capacity to yield high-purity polymer (>95%). Oxidising agents like sodium hypochlorite effectively break down proteins, lipids, carbohydrates, and nucleic acids. However, this method is linked to notable limitations, especially the decrease in molecular weight, which compromises polymer properties.^142^ Modifications like combining sodium hypochlorite with chloroform mitigate this degradation and facilitate recovery.^143^ Similarly, alkaline treatments using KOH and NaOH disrupt cell membranes, although their efficiency depends on the type of culture, with lower efficiency observed in mixed cultures.^72^ Methods based on acids, such as sulfuric acid, provide a more cost-effective option with minimal PHA degradation but require precise optimisation to minimise structural degradation.^144^ Despite their efficiency, environmental concerns and potential toxicity limit their environmental viability. In contrast, enzymatic extraction has gained attention as a more sustainable approach that provides high extraction efficiency and maintains polymer integrity. Enzymes such as proteases, nucleases, and lysozymes selectively hydrolyse cellular impurities, yielding high-purity polymers. For example, the use of *Aspergillus awamori*-derived enzymes has been reported to achieve up to 98% yield and 97% purity without the use of chemical solvents. However, the enzymatic method continues to encounter significant constraints due to the high cost of enzymes for large-scale production.^145^ Hybrid strategies combining enzymatic digestion with surfactants (SDS) and chelating agents (EDTA) have been evaluated to increase performance; additional optimisation is needed to achieve a balance between cost and efficiency.^42^ Biological recovery methods, which employ whole organisms to degrade cellular components, provide an eco-friendly alternative to maintain the polymer characteristics. However, these methods are typically slower and less controllable, limiting their large-scale applicability,^146^ particularly in industrial settings where time and precision are critical for efficiency and cost-effectiveness. Advanced strategies utilising genetically modified microbial systems, including bacteriophage-induced lysis systems and secretion-based recovery mechanisms, provide potential to simplify post-processing operations. For example, engineered *Alcanivorax borkumensis* strains have shown the capability to secrete extracellular PHAs under controlled growth conditions.^147^ Despite these advantages, these methods are still in the early stages of development and need further assessment for industrial applications.

Mechanical cell disruption methods depend on mechanical forces to disrupt cell structures and liberate intracellular PHAs, serving as a substitute for chemically intensive processes. Techniques like microbead milling produce strong shear forces capable of effectively disrupting cell walls, achieving 100% recovery and 94% purity within 2 hours when combined with SDS.^148^ High-pressure homogenisation (HPH) also creates high shear stress and turbulence in small spaces, which make it possible to achieve a high recovery rate of 98% and a purity rate of 95% for P(3HB).^149^ Ultrasound-assisted disruption, which employs high-frequency sound waves, has also been investigated, particularly when integrated with chemical treatments to increase recovery performance and potentially modify polymer properties.^150^ A patented integrated approach combining bead milling, ultrasound, pH shifts, and surfactants has demonstrated high efficiency at low cost.^151^

The high energy demand of mechanical methods, combined with the environmental concerns associated with chemical solvents, underscores the need for more sustainable recovery strategies, such as biologically driven approaches that utilise genetic and metabolic engineering to enhance product release and minimise environmental impact. Biologically driven approaches, including cell disruption *via* engineered fragility, yeast-surface engineering, and protein-mediated secretion systems, are gaining attention. These strategies leverage genetic and metabolic engineering to facilitate intracellular product release, thereby reducing downstream processing requirements.^152^ However, their practical implementation remains limited by challenges related to process control and scalability, such as the difficulty in maintaining consistent product quality and the high costs associated with scaling up these biotechnological processes.

Cell-fragility-based approaches represent another promising strategy, exploiting weakened cell structures for enhanced PHA release. Pretreatment methods involving solvents, enzymes, or chemical agents can increase membrane permeability, while the inherently high intracellular PHA accumulation (typically 60–80% of dry cell weight) further promotes cell lysis.^153^ For example, osmotic lysis using alkali or detergents has been successfully applied in halophilic microbes like *H. mediterranei* and recombinant *E. coli* for the recovery of P(3HB-*co*-3HV).^154^ Although these approaches are effective, they require careful optimisation to balance efficiency with polymer integrity. Basically, mechanical and cell-fragility-based methods provide effective alternatives to conventional chemical extraction; however, their large-scale application is constrained by energy demands, cost, and process complexity. Consequently, future research should prioritise the development of integrated and biologically assisted recovery systems that minimise environmental impacts while maintaining high recovery efficiency and polymer quality.^41^

### Innovative biological techniques

Mealworm-assisted extraction has been explored as a biological approach to PHA recovery, in which mealworms are fed freeze-dried bacterial cells and selectively digest non-PHA cellular components, excreting faecal pellets enriched with PHB granules. The recovered polymer maintains its native spherical morphology and can attain near-complete purity following additional washing steps using water, heat, and detergents, including 1% sodium dodecyl sulphate (SDS), which has been shown to further enhance purity up to 100%. Moreover, this method offers an added sustainability advantage by supporting a circular bioeconomy, as the mealworms themselves can be repurposed as protein-rich fish feed after processing.^41,145^ While innovative, such approaches remain at an early stage and require further validation in terms of scalability, consistency, and regulatory acceptance.

### Purification

Purification constitutes a key downstream stage in PHA production, as it directly determines polymer quality, application suitability, and overall process cost. After extraction, the composition of residual contaminants is significantly influenced by the recovery technique: solvent-based extraction often co-isolates lipids and pigments, whereas chemical digestion typically leaves protein residues. Consequently, purification strategies must be carefully chosen based on both impurity composition and intended application requirements, such as acceptable endotoxin levels and molecular-weight specifications.^155^ Additionally, the purification and optimal extraction techniques are influenced by the type of PHA (such as mcl-PHA or P(3HB)), the specific microbial strain used, the intracellular PHA content, and the cultivation conditions.^42^ Comparative assessments of purification strategies are based on cost and environmental sustainability. Solvent-based purification can yield high polymer purity but is constrained by toxicity, high cost, and environmental issues. In contrast, surfactant hypochlorite treatments are effective for removing cellular debris but can cause polymer degradation, reducing molecular weight. Alkaline treatments, on the other hand, have been found to be a better choice for the environment and the wallet, as they strike a balance between removing contaminants and keeping polymers intact.^156^ No single purification method is universally optimal. Instead, the choice depends on achieving a balance between product quality, economic viability and environmental sustainability. This emphasises a critical limitation in PHA commercialisation: downstream processing remains a major contributor to overall production costs and sustainability challenges.

### Biodegradability of PHA and its benefit to the environment

**Fig. 6:**
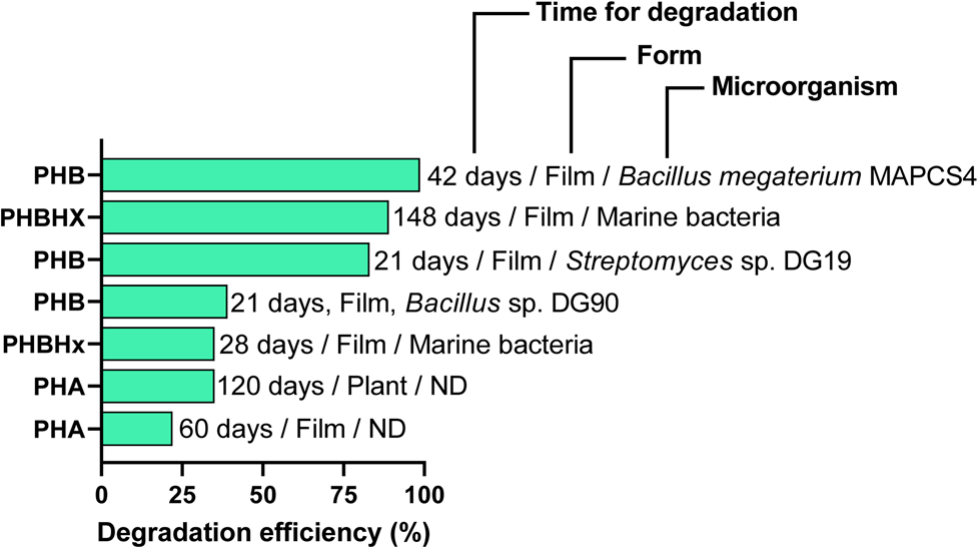
Biodegradation efficiency of polyhydroxyalkanoates by different microbial agents. (PHB, polyhydroxybutyrate; PHBHx, (poly(3-hydroxybutyrate-co-3-hydroxyhexanoate)); PHA, polyhydroxyalkanoates; ND, no data; [figure drawn using data from ref. 161–165].

**Fig. 7:**
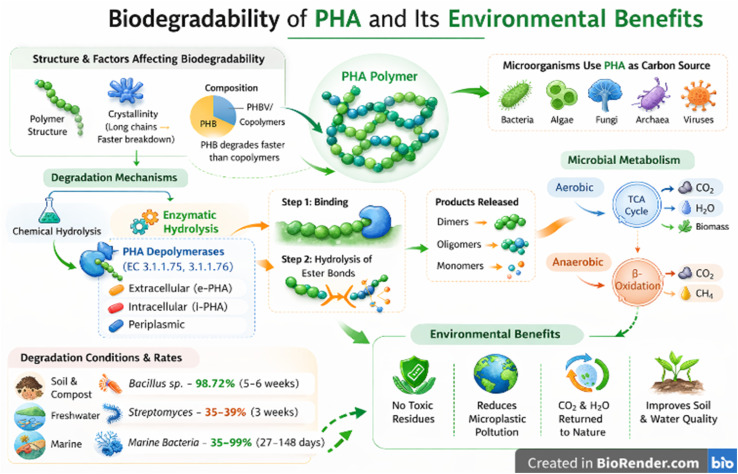
PHA biodegradability and its environmental effects.

The biodegradation of PHAs is influenced by both intrinsic polymer characteristics (*e.g.*, monomer composition, crystallinity, and molecular weight) and surrounding environmental conditions, like temperature, microbial diversity, and nutrient availability.^157^ Enzymatic activities and chemical hydrolysis are the major mechanisms employed to cause degradation and surface erosion.^158^ Microorganisms, including bacteria, fungi, algae, and archaea, utilise PHAs as carbon and energy sources, with depolymerase enzymes specifically and selectively binding to the R-configured monomers of PHA.^159^ The resulting oligomers and monomers are further incorporated into microbial metabolic pathways, where PHAs are broken down.^159^ However, the biodegradation performance of PHAs varies from that of partially biodegradable plastics because no residual particles persist. The intrinsic properties of polymers and environmental conditions influence the process of biodegradation. For example, short-chain-length PHAs, such as PHB, generally degrade faster than copolymers like PHBV due to differences in crystallinity and structural organization.^160^ Similarly, environmental conditions strongly influence degradation rates: marine ecosystems, freshwater, soil and composts have all been studied by researchers for PHA degradation.^157^ For instance, *Streptomyces* sp. DG19 and *Bacillus* sp. DG90 achieved up to 35% and 39% PHB degradations within 3 weeks; *Bacillus megaterium* MAPCS4 achieved up to 98.72% PHB degradation within 5–6 weeks.^161,162^ The marine bacterial degradation rate of PHBHx at 27 °C and PHBHx at 23 °C ranges from 35% in 28 days to 99% over extended periods (148 days), as shown in Fig. 6 and 7.^163,164^

The homopolymer PHB degrades more quickly than copolymers, such as PHBV, as the rate at which polymers degrade is dependent on their composition.^166^ When PHA degrades, ester bonds are hydrolysed by PHA depolymerases (EC 3.1.1.75, EC 3.1.1.76) to produce dimers, oligomers, and monomers that microbes assimilate.^165^ Depolymerases are classified based on their localisation and substrate specificity. Extracellular depolymerases (e-PHA depolymerases) degrade extracellular crystalline granules. Intracellular depolymerases (i-PHA depolymerases) mobilise intracellular PHA reserves, while periplasmic depolymerases support predatory or metabolic processes in certain bacteria. For example, *P. lemoignei* secretes PhaZ7, an extracellular depolymerase that specifically attacks native PHB granules,^167^ while *B. bacteriovorus* employs periplasmic depolymerases during predation.^168^ Substrate selectivity distinguishes scl-PHA depolymerases, which preferentially hydrolyse PHB into 3-hydroxybutyrate, from medium-chain-length (mcl-PHA) depolymerases, which act on polymers such as poly(3-hydroxyhexanoate).^169^ Extracellular depolymerases initiate degradation by binding to polymer surfaces and hydrolysing ester bonds, as shown in Fig. 7,^170^ whereas intracellular enzymes mobilise stored PHAs within cells. Notably, substrate specificity varies, with enzymes selectively targeting either short-chain-length or medium-chain-length PHAs, further influencing degradation efficiency.^171^ The short-chain-length PHA mechanism for degradation starts from the non-crystalline region to the crystalline on the polymer surface, while non-crystalline medium-chain-length polymers undergo internal degradation.^42^ Following enzymatic hydrolysis, PHA monomers enter microbial metabolic pathways. Under aerobic conditions, they are funnelled into the TCA cycle, yielding CO_2_, water, and biomass, as shown in Fig. 7. In anaerobic environments, metabolism proceeds *via* the β-oxidation pathway, producing CO_2_ and methane.^172^ However, compared to conventional plastics, PHAs offer a significantly improved environmental profile, as they degrade into non-toxic by-products that reintegrate into natural biogeochemical cycles, aligning with green chemistry principles.^173^ This degradation process not only reduces pollution but also supports ecosystem health by returning nutrients to the soil and water systems.^174–180^

### Comparison of PHA with conventional plastics

Conventional plastics are mainly petroleum-derived synthetic polymers produced from non-renewable resources, like crude oil, natural gas, and coal.^186^ Their extensive use is primarily due to their advantageous properties, including low density, strong durability, resistance to chemical degradation, and excellent barrier performance against moisture and gases.^181–185^ These characteristics, together with low production costs and well-established industrial infrastructure, have ensured their market prevalence.^187^ However, their persistence in the environment and resistance to biodegradation have resulted in significant environmental challenges, such as the accumulation of micro- and nanoplastics and associated impacts on ecosystems and human health.^188^ In contrast, PHAs constitute a group of bio-based and biodegradable polymers produced by microorganisms from renewable carbon sources. Unlike conventional plastics, PHAs can be completely degraded by microbial processes into carbon dioxide, water, and biomass under appropriate environmental conditions, consequently reducing their persistent environmental accumulation. They possess improved material functionality, making them suitable for food packaging, in contrast to polypropylene.^189^ Biobased polymers are termed “eco-friendly plastics”, as they contain no harmful substances, are easily recyclable, require less energy, and support sustainability.^190^

This biodegradability makes PHAs promising candidates within a circular economy, where material recovery and environmental compatibility are emphasised. Despite these environmental benefits, PHAs currently face significant limitations when compared to conventional plastics. Economically, their production cost remains substantially higher, primarily attributed to costly feedstocks, fermentation processes, and downstream processing steps. In terms of material performance, certain PHA types (PHB) exhibit brittleness and a limited processing window, which limit their application relative to widely used polymers, such as polypropylene. Although copolymer formation (PHBV, PHBHx) improves flexibility and toughness, such modifications often raise production complexity and overall cost.^189^ Furthermore, when compared with alternative biopolymers, like polylactic acid (PLA) or starch-based blends, PHAs offer enhanced biodegradability under environmental conditions but remain limited in terms of industrial scalability and market penetration. This highlights a critical trade-off: while conventional plastics perform strongly in terms of cost-efficiency and consistent performance and other bioplastics benefit from established supply networks, PHAs offer exceptional environmental advantages but require further technological and economic optimisations to compete effectively.^41^ The comparison underscores that PHAs are not yet complete substitutes for all conventional plastics but are better regarded as complementary materials for applications where biodegradability and sustainability are prioritised over cost and mechanical properties.

### Application of PHAs as alternatives to plastics

Due to their biodegradability, biocompatibility, and different material properties, PHAs have gained considerable attention as eco-friendly substitutes to traditional plastics across industries. Their versatility arises from the variety of monomer compositions, which allows for the tailoring of their mechanical and thermal properties for specific applications, such as packaging, agricultural films, and medical devices. Scl-PHAs, such as PHB, have a high degree of crystallinity but a low tensile strength. However, their inherent brittleness and low tensile strength limit their durability, restricting their use mainly to rigid, single-use products, such as cutlery and disposable packaging. In contrast, mcl-PHAs, like copolymers P(3HB-*co*-3HV) and PHBHHx, display enhanced flexibility, lower crystallinity, and improved impact resistance. These properties make them better suited for applications demanding flexibility, including biomedical devices and flexible packaging films. Nonetheless, these improved material properties often come at the expense of increased production complexity and cost,^191^ which can limit their widespread adoption in various industries, including packaging and biomedicine.

#### Use in packaging

Packaging materials are required to offer sufficient barrier performance properties against oxygen, moisture, and contaminants while maintaining mechanical integrity.^192^ In comparison with traditional plastics, such as polyethylene and polypropylene, PHAs provide the benefit of full biodegradability; however, their barrier and processing properties are highly dependent on monomer composition. For instance, PHB shows very low oxygen permeability (roughly 2–10 cc mm m^−2^ 24 h^−1^ atm^−1^), making it suitable for food preservation.^193^ However, its inherent brittleness and limited processing range limit its suitability for large-scale packaging applications, especially for injection moulding processes. To address these limitations, copolymers such as P(3HB-*co*-3HV) are commonly used, as they enhance flexibility and processing performance.^194^ Despite these improvements, PHA-based packaging still faces challenges in competing with conventional plastics in terms of cost, scalability, and processing efficiency.

#### Use in agriculture

**Fig. 8:**
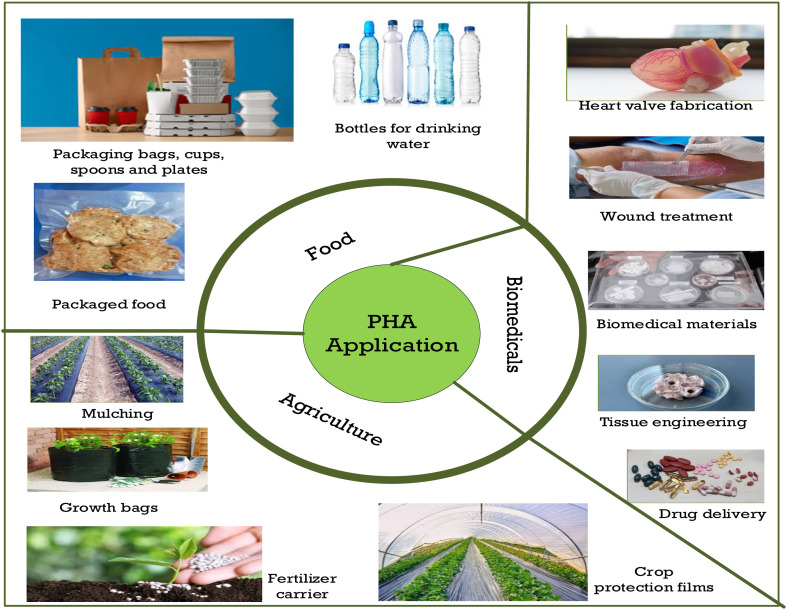
Application of polyhydroxyalkanoates (PHA).

In the agricultural sector, PHAs provide considerable environmental advantages due to their biodegradability in soil environments. They are widely investigated as alternatives to conventional-based mulch films, which persist in the environment and lead to soil pollution and landfill buildup. PHA-based mulch films can improve soil quality, maintain soil moisture, inhibit weed growth, and reduce pollution, thereby supporting sustainable agricultural practices.^195^ However, while PHAs provide clear ecological advantages, their adoption remains limited by economic and functional constraints. Conventional plastics, such as low-density polyethylene (LDPE) and high-density polyethylene (HDPE), dominate the market due to their durability. In contrast, PHA-based materials may exhibit lower mechanical strength and higher degradation rates, which can reduce their functionality and shorten their service life.^196^ Beyond mulch films, PHAs have also been applied in slow-release systems for fertilisers, herbicides, and pesticides,^197^ as well as in biodegradable plant growth bags. These applications demonstrate their potential to minimise environmental toxicity, reduce root deformation, speed plant growth, make plants more resistant to infections, and improve agricultural productivity, as shown in Fig. 8.^198^ However, industrial-scale implementation requires further optimisation to balance degradation rates with functional performance and lower production costs.

#### Uses in medicine

Polyhydroxyalkanoates (PHAs), particularly medium-chain-length PHAs (mcl-PHAs), have gained considerable attention in biomedical applications due to their excellent compatibility, degradability, and modifiable mechanical properties.^42^ Compared to conventional synthetic polymers, such as polyethylene or polylactic acid (PLA), PHAs provide a clear advantage in that they degrade into non-toxic by-products that can be assimilated by the body, minimising the risk of chronic inflammation and undesirable immune reactions. One emerging application of PHAs is in diagnostic systems. For instance, polyhydroxybutyrate (PHB) has been documented to selectively engage with breast cancer cells through surface proteins while demonstrating negligible interaction with normal cells, indicating its potential as a target-specific diagnostic agent.^199^ While these applications show promise, they are still mostly in the experimental stage and need more testing before they can be used in real life. In tissue engineering, PHAs show considerable potential due to their structural versatility. Copolymers such as PHBV and flexible mcl-PHAs have been extensively studied as scaffolds for skin regeneration, while PHBHHx, often combined with granule-binding protein (PhaP), has shown effectiveness in bone and cartilage tissue engineering.^159,182^ Compared to traditional biomaterials, PHAs offer enhanced biodegradability and biological activity; however, constraints such as reduced mechanical strength and limited scalability in processing can restrict their widespread clinical use. Additionally, the flexibility of mcl-PHAs makes them appropriate for cardiovascular applications, including heart valve fabrication. Copolymers such as P(3HB-*co*-4HB) exhibit improved elasticity and lower crystallinity, making them promising candidates for vascular grafts and soft tissue engineering.^200^ However, when compared to widely used materials like medical-grade polyurethanes, PHAs still require additional optimisation for sustained mechanical stability and regulatory requirements. PHAs have also been widely investigated in drug delivery due to their ability to degrade in a regulated manner, allowing controlled release of drugs.^80^ This property, combined with their non-toxic degradation products, provides a significant advantage over some synthetic polymers that may generate harmful residues, particularly in applications such as drug delivery and tissue engineering, where safety and efficacy are paramount. Although PHAs demonstrate considerable potential for prospective use in tissue engineering and regenerative therapies, medical diagnostics and drug delivery, further investigation is required to enhance the material characteristics, improve cost-efficiency, and validate their long-term biocompatibility and functionality in medicine.

### Problems restricting PHA commercialization

PHAs have gained considerable attention as promising eco-friendly biopolymers, owing to their biodegradability, biocompatibility, and bio-based origin.^73^ However, economic limitations primarily constrain their implementation on an industrial scale. Current estimates indicate that PHAs (4–6 USD per kg; typically, 4–7 USD per kg) remain considerably more costly than conventional plastics, such as polyethylene and polypropylene (1–2 USD per kg), as well as other bioplastics, such as polylactic acid (PLA) (approximately 2.0–3.7 USD per kg).^201,202^ This represents a cost disparity of approximately three-to four-fold at the resin level, highlighting a significant economic barrier to their large-scale commercialisation. These cost differences are largely driven by two major contributors: substrate cost, which accounts for approximately 50–60% of total production costs, and downstream recovery and purification stages, contributing an additional 30–50%.^201,202^ Relative to traditional plastics, which leverage optimised large-scale petrochemical systems, PHA production remains biologically intricate and less industrially mature. Moreover, achieving a balance between high cell density and maximal polymer accumulation remains a persistent technical challenge, thereby reducing process efficiency. Despite these limitations, emerging studies suggest that the economic gap can be gradually narrowed. The utilisation of inexpensive and waste-based substrates (like agricultural residues and waste oils) has shown potential to significantly reduce substrate costs while maintaining competitive yields. Concurrently, advances in process optimisation, continuous fermentation, and improved downstream processing are beginning to mitigate scalability limitations.^203^ However, when compared to other bioplastics like PLA, which already benefit from established supply chains and industrial integration, PHAs fall short in terms of market readiness and cost competitiveness. This analysis shows that PHAs have a better strategic position than low-cost commodity plastics, which is an important finding. PHAs are more realistically suited for high-value, application-specific markets where their unique characteristics, like complete biodegradability and, in some cases, marine degradability, provide clear advantages. This is evident in current market trends, where PHAs are experiencing increased adoption in sectors, for example, packaging, agriculture, and biomedicine. The global PHA market, currently estimated at 70–120 million USD, is expected to grow to 170–265 million USD by 2030, with annual growth rates ranging from 8% to 16% depending on application and region.^204,205^ From an analytical perspective, the future viability of PHAs will be determined not only by reducing production costs but also by re-evaluating their role within the broader materials economy. Instead of acting as direct substitutes for conventional plastics, PHAs are better positioned as complementary materials for specialised, regulatory-driven, and environmentally critical applications.^201^ This repositioning constitutes a significant step toward aligning technological development with practical industrial and economic limitations, as it allows for the integration of PHAs into existing systems while addressing sustainability goals and regulatory requirements.

### Recent advances and future perspective

Despite their potential as viable alternatives to traditional plastics, PHAs face market limitations due to a lack of regulatory support, high production costs, and low process efficiency.^206^ Advancing a circular bioeconomy is critical for sustainable development, as it prioritises optimal resource use, low-emission technologies, and the incorporation of biotechnological processes. Enhancing public awareness, implementing legislative measures on plastics, and increasing consumer awareness of the environmental impacts of conventional plastics are essential.^207,208^ The adoption of biodegradable materials could be accelerated through focused policy interventions intended to restrict fossil-based plastic production.

Despite their advantages, the management of end-of-life scenarios for both biobased and conventional plastics remains a significant challenge. Improper disposal or co-processing with non-biodegradable materials may compromise environmental benefits and contribute to microplastic pollution.^209^ Furthermore, emerging studies indicate that biodegradation intermediates and substrate utilisation pathways may pose unforeseen ecological risks, highlighting the need for continuous monitoring and comprehensive environmental assessments.^210,211^

From an economic perspective, both upstream production and downstream recovery processes demand concurrent optimisation, as each stage directly influences the total production costs. Substrate costs remain a major contributor to production costs. While both monocultures and mixed cultures can utilise low-cost residual substrates to reduce costs, mixed cultures often exhibit lower PHA yields.^212,213^ Downstream processing, especially extraction, continues to represent a major limitation.^214^ Recent advancements, such as the use of extremophiles capable of growing under non-sterile conditions, provide opportunities for lowering costs and process simplification. However, preserving polymer purity and consistent structural properties is still problematic due to the sensitivity of PHA properties to downstream processing conditions,^215^ which can lead to variations in product quality and performance in applications, such as biodegradable plastics and medical devices. Although still emerging, the application of continuous fermentation systems, coupled with AI-driven bioreactor control, presents a promising strategy for improving process efficiency and reducing energy input. AI-assisted modelling and optimisation can significantly reduce reliance on empirical experimentation, thereby accelerating process development and lowering associated costs.^201^

Future research should prioritise the development of cost-effective and environmentally sustainable extraction methods, as well as strategies to enhance yield through metabolic and genetic engineering. Approaches such as cell-wall modification *via* gene knockouts^216^ or pathway engineering to enable extracellular PHA secretion^217^ present promising opportunities. Extracellular accumulation could significantly simplify recovery processes and overcome intracellular storage limitations.

Advances in strain engineering have also demonstrated notable progress. For instance, evolutionary adaptation strategies have improved substrate utilisation efficiency, as observed in engineered *E. coli* strains with enhanced sucrose metabolism that achieved substantial PHB accumulation.^76^ Similarly, targeted modification of metabolic pathways, such as the deletion of fadA and fadB genes in β-oxidation pathways, reduces metabolic competition and enhances polymer yield.^218^ Additionally, alternative cell-division mechanisms have been proposed to further increase intracellular polymer accumulation.^219^

Recent developments in CRISPR/Cas9 and CRISPR interference (CRISPRi) technologies provide powerful tools for precise genetic and transcriptional regulation of PHA biosynthetic pathways. These approaches enable multiplex gene editing, facilitating improved control over polymer composition and monomer distribution.^220^ Given that variability in PHA synthase (PhaC) activity often results in inconsistent molecular weight and polymer architecture, targeted regulation of PhaC expression represents a promising strategy for achieving consistent product quality.^219,220^

## Conclusion

PHAs represent an emerging group of biopolymers with the potential to contribute significantly to the transition away from fossil-based plastics. Their degradability, biological compatibility, and adaptability to renewable and waste-derived feedstocks position them as suitable alternatives to conventional plastics for sustainable material innovation. However, this study demonstrates that the widespread replacement of conventional plastics with PHAs remains unlikely in the near term due to persistent economic and technical limitations. Analytical evaluation of existing studies indicates that the primary bottleneck is not a single factor, but the combined effects of substrate cost, process efficiency, and downstream recovery. While low-cost substrates, mixed microbial cultures, and metabolic engineering strategies present significant potential for cost reduction, these approaches often introduce trade-offs in yield, process stability, or material quality. Similarly, advances in strain engineering and process optimisation have improved productivity; yet challenges related to scalability, polymer consistency, and efficient extraction remain unaddressed. These findings suggest that future progress will depend on integrated, system-level optimisation rather than isolated technological improvements.

Notably, this review positions PHAs not as universal substitutes for conventional plastics, but as high-performance, application-specific materials suited to applications in which biodegradability and environmental performance provide clear advantages. This perspective aligns more realistically with current economic and technological limitations and highlights the need to focus on specialised applications over large-scale commodity substitutions. Beyond technological and economic considerations, the large-scale deployment of PHAs will rely on the implementation of consistent and standardised biodegradability benchmarks, along with supportive policy frameworks. Existing standards, like EN 13432, ISO 17088/18606, and ASTM D6400, define industrial composability, while additional standards (EN 17033 and ISO 17556) cover soil biodegradation and ecotoxicity. However, variability across environments and inadequate infrastructure remain major obstacles. At the same time, emerging regulatory frameworks, such as the EU Single-Use Plastics Directive and extended producer responsibility schemes, are increasingly influencing material choice and waste management. Optimising PHA development with these regulatory and environmental requirements will be critical to ensuring its effective integration into waste-management systems. Overall, the future competitiveness of PHAs will depend on a coordinated approach that integrates technological innovation, economic feasibility, and regulatory alignment. Rather than focusing solely on production efficiency, research efforts should emphasise system integration, application-driven material design, and lifecycle performance. Such a shift in perspective is critical to bridging the gap between laboratory-scale advances and real-world implementation, ultimately enabling PHAs to play a targeted yet impactful role within a circular bioeconomy.

## Author contributions

Konjerimam Ishaku Chimbekujwo: investigation, writing – original draft, writing – review and editing. Udeme Joshua Josiah Ijah: supervision, Oluwafemi Adebayo Oyewole: conceptualization, supervision, writing – review and editing. Olabisi Peter Abioye: supervision, Evans Chidi Egwim: conceptualization, supervision, Mattheus Victor Maso Lacorte Silva: data curation, formal analysis, writing – review and editing. Naga Raju Maddela: writing – review and editing, and Jonas Contiero: supervision, writing – review and editing.

## Conflicts of interest

The authors declare no conflicts of interest.

## Data Availability

Data sharing is not applicable to this article as no new data set was generated or analyzed in this review article.
