# Correction: Polyhydroxyalkanoates as multifaceted biopolymers: an eco-friendly alternative to conventional plastics

**DOI:** 10.1039/d6ra90065h

**Published:** 2026-07-06

**Authors:** Konjerimam Ishaku Chimbekujwo, Udeme Joshua Josiah Ijah, Oluwafemi Adebayo Oyewole, Olabisi Peter Abioye, Evans Chidi Egwim, Matheus Victor Maso Lacôrte e Silva, Naga Raju Maddela, Jonas Contiero

**Affiliations:** a Department of Microbiology, Federal University of Technology Minna Nigeria; b Department of Microbiology, Modibbo Adama University Yola Nigeria; c Institute of Biosciences, Institute for Research in Bioenergy, Sao Paulo State University Rio Claro SP Brazil Konjechimbe@mau.edu.ng; d Department of Biochemistry, Federal University of Technology Minna Nigeria; e Departamento De Ciencias Biológicas, Facultad De Ciencias de la Salud, Universidad Técnica De Manabí Portoviejo-130105 Ecuador

## Abstract

Correction for ‘Polyhydroxyalkanoates as multifaceted biopolymers: an eco-friendly alternative to conventional plastics’ by Konjerimam Ishaku Chimbekujwo *et al.*, *RSC Adv.*, 2026, **16**, 29399–29422, https://doi.org/10.1039/D6RA02201D.

The authors regret that the name of one of the authors (Matheus Victor Maso Lacôrte e Silva) was shown incorrectly in the original article. The corrected author list is as shown above.

The Royal Society of Chemistry apologises for these errors and any consequent inconvenience to authors and readers.

